# Supplementary material for: Bacon, Brownie, or Broccoli? Beliefs about Stress-Relieving Foods and Their Relationship to Orthorexia Nervosa
Source: Nutrients. 2022 Sep 6;14(18):3673. doi: 10.3390/nu14183673 (PMC9505357; doi:10.3390/nu14183673)
Supplement: Supplementary file 1 [file nutrients-14-03673-s001.zip › Table_S4_First PCA.pdf]

Table S4 First Principal Component Analysis

Obliquely rotated factor loadings for 23 food items

Principal Components Analysis

food item	Factor 1	Factor 2	Factor 3
bread	**0.53**	**0.40**	**0.27**
pasta	**0.39**	**0.45**	**0.36**
potatoes	**0.48**	**0.11**	**0.48**
rice	**0.46**	**0.19**	**0.46**
butter	0.61	0.34	0.18
couscous	0.21	0.18	0.54
legumes	0.37	0.02	0.62
eggs	0.79	0.09	0.21
ready meals	0.28	0.57	-0.01
fish	0.55	-0.05	0.36
meat	0.75	0.21	-0.07
vegetables	0.08	0.02	0.84
cheese	0.49	0.31	0.27
salty nibbles	0.13	0.71	0.13
cake	0.03	0.79	0.11
milk products	0.64	0.22	0.20
cereals	0.35	0.49	0.23
nuts	-0.01	0.10	0.62
fruits	0.22	0.14	0.62
salad	0.34	-0.05	0.64
sweet spread	0.33	0.63	0.09
soups	0.06	0.15	0.67
sweets	0.00	0.79	0.00
eigen values	4.09	3.53	4.07
% of variance	0.18	0.15	0.18

Mean item complexity = 1.6

Test of the hypothesis that 3 components are sufficient.

The root mean square of the residuals (RMSR) is 0.06 with the empirical chi square 364.68 with prob < 1.5e-13

Fit based upon off diagonal values = 0.96

food items with loadings greater or equal 0.4 on more than one factor are displayed in bold type
