# Supplementary material for: Bacon, Brownie, or Broccoli? Beliefs about Stress-Relieving Foods and Their Relationship to Orthorexia Nervosa
Source: Nutrients. 2022 Sep 6;14(18):3673. doi: 10.3390/nu14183673 (PMC9505357; doi:10.3390/nu14183673)
Supplement: Supplementary file 1 [file nutrients-14-03673-s001.zip › Table_S5_Final PCA.pdf]

Table S5 Final Principal Component Analysis

Obliquely rotated factor loadings for 19 food items

Principal Component Analysis

Food item	Factor 1	Factor 2	Factor 3
eggs	**0.81**	0.10	0.23
meat	**0.75**	0.22	-0.06
milk products	**0.66**	0.23	0.22
butter	**0.60**	0.35	0.20
fish	**0.59**	-0.03	0.37
cheese	**0.52**	0.29	0.30
cake	0.02	**0.79**	0.11
sweets	0.04	**0.79**	0.03
salty nibbles	0.12	**0.72**	0.14
sweet spread	0.35	**0.63**	0.11
ready meals	0.24	**0.58**	-0.02
cereals	0.31	**0.51**	0.22
vegetables	0.09	0.01	**0.85**
soups	0.06	0.16	**0.67**
salad	0.33	-0.06	**0.65**
fruits	0.25	0.13	**0.64**
nuts	-0.02	0.11	**0.63**
legumes	0.35	0.03	**0.62**
couscous	0.19	0.16	**0.55**
eigen values	3.26	3.18	3.55
% of variance	0.17	0.17	0.19

Mean item complexity = 1.4

Test of the hypothesis that 3 components are sufficient.

The root mean square of the residuals (RMSR) is 0.07 with
the empirical chi square 256.62 with prob < 1.8e-12

Fit based upon off diagonal values = 0.95

Note. food items with loadings greater or equal 0.4 are displayed in bold type
